# Supplementary material for: Structure of E3 ligase E6AP with a proteasome-binding site provided by substrate receptor hRpn10
Source: Nat Commun. 2020 Mar 10;11:1291. doi: 10.1038/s41467-020-15073-7 (PMC7064531; doi:10.1038/s41467-020-15073-7)
Supplement: Supplementary file 3 — Reporting Summary [file 41467_2020_15073_MOESM3_ESM.pdf]

nature research

Corresponding author(s): NCOMMS-19-32780A

Last updated by author(s): Feb 3, 2020

Reporting Summary

Nature Research wishes to improve the reproducibility of the work that we publish. This form provides structure for consistency and transparency in reporting. For further information on Nature Research policies, see Authors & Referees and the Editorial Policy Checklist .

Statistics

For all statistical analyses, confirm that the following items are present in the figure legend, table legend, main text, or Methods section.

n/a	Confirmed	
☐	☒	The exact sample size (*n*) for each experimental group/condition, given as a discrete number and unit of measurement
☐	☒	A statement on whether measurements were taken from distinct samples or whether the same sample was measured repeatedly
☒	☐	The statistical test(s) used AND whether they are one- or two-sided<br>*Only common tests should be described solely by name; describe more complex techniques in the Methods section.*
☒	☐	A description of all covariates tested
☒	☐	A description of any assumptions or corrections, such as tests of normality and adjustment for multiple comparisons
☐	☒	A full description of the statistical parameters including central tendency (e.g. means) or other basic estimates (e.g. regression coefficient) AND variation (e.g. standard deviation) or associated estimates of uncertainty (e.g. confidence intervals)
☒	☐	For null hypothesis testing, the test statistic (e.g. *F*, *t*, *r*) with confidence intervals, effect sizes, degrees of freedom and *P* value noted<br>*Give P values as exact values whenever suitable.*
☒	☐	For Bayesian analysis, information on the choice of priors and Markov chain Monte Carlo settings
☒	☐	For hierarchical and complex designs, identification of the appropriate level for tests and full reporting of outcomes
☒	☐	Estimates of effect sizes (e.g. Cohen's *d*, Pearson's *r*), indicating how they were calculated

Our web collection on statistics for biologists contains articles on many of the points above.

Software and code

Policy information about availability of computer code

Data collection	Bruker TopSpin
Data analysis	NMRpipe, XEASY, TALOS+, XPLOR-NIH, PROCHECK-NMR, MOLMOL, PyMOL

For manuscripts utilizing custom algorithms or software that are central to the research but not yet described in published literature, software must be made available to editors/reviewers. We strongly encourage code deposition in a community repository (e.g. GitHub). See the Nature Research guidelines for submitting code & software for further information.

Data

Policy information about availability of data

All manuscripts must include a data availability statement. This statement should provide the following information, where applicable:

- Accession codes, unique identifiers, or web links for publicly available datasets
- A list of figures that have associated raw data
- A description of any restrictions on data availability

Atomic coordinates for RAZUL:AZUL have been deposited in the Protein Data Bank (PDB) with accession number 6U19. Chemical shift assignments have been deposited in the Biological Magnetic Resonance Data Bank (BMRB) with accession number 27875.

Field-specific reporting

Please select the one below that is the best fit for your research. If you are not sure, read the appropriate sections before making your selection.

☒ Life sciences ☐ Behavioural & social sciences ☐ Ecological, evolutionary & environmental sciences

Life sciences study design

All studies must disclose on these points even when the disclosure is negative.

Sample size	No statistical methods were applied to pre-determined sample sizes. During structure calculations of the protein complex, 50 random linear structures were used as starting structures. After simulated annealing and energy minimization steps, the 15 lowest energy structures were chosen for visualization and statistical analyses.
Data exclusions	No data were excluded from the analysis.
Replication	Biochemical experiments, including protein purification, and SDS-PAGE were done in biological replicates of greater than 10 times. Biophysical experiments, including SRP, ITC and CD were done in triplicate. Experiments utilizing mammalian cells were performed in a minimum of two replicates. All attempts of replication were successful.
Randomization	This study did not allocate experimental groups thus no randomization was required for the reported experiments.
Blinding	Blinding was not required for the reported experiments, because all functional and structural data were analyzed using the same methods.

Reporting for specific materials, systems and methods

We require information from authors about some types of materials, experimental systems and methods used in many studies. Here, indicate whether each material, system or method listed is relevant to your study. If you are not sure if a list item applies to your research, read the appropriate section before selecting a response.

Materials & experimental systems

n/a	Involved in the study	
☐	☒	Antibodies
☐	☒	Eukaryotic cell lines
☒	☐	Palaeontology
☒	☐	Animals and other organisms
☒	☐	Human research participants
☒	☐	Clinical data

Methods

n/a	Involved in the study	
☒	☐	ChIP-seq
☒	☐	Flow cytometry
☒	☐	MRI-based neuroimaging

Antibodies

Antibodies used	Primary antibodies used were: beta-actin (Cell Signaling Technologies 4970), Cyclophilin B (Abcam ab178397), E6AP (MilliporeSigma E8655), HA-tag (MilliporeSigma H6908), myc-tag (Cell Signaling Technologies 2278), Rpn10 (Novus Biologicals NBP2-19952), Rpn2 (Bethyl Laboratories A303-851A), Rpn13 (Abcam ab140515), Rpn11 (Cell Signaling Technologies 4197), Rpn8 (abcam ab140428), and ubiquitin (MilliporeSigma MAB1510). Secondary antibodies used were: Rabbit HRP (Life technologies A16110) and mouse HRP (MilliporeSigma A9917).
Validation	Information of the antibody validation is available through manufacturer's online database. Further validation was done on the antibody against the control in the reported experiments.

Eukaryotic cell lines

Policy information about cell lines

Cell line source(s)	HCT116 were purchased from American Type Culture Collection (ATCC CCL-247). HCT116 deltaRAZUL cells were generated via CRISPR-Cas9 methodology.
Authentication	None of the commercial cell lines used were further authenticated. HCT116 deltaRAZUL cells were validated by sequencing PCR-amplified genomic DNA in addition to Western blotting.
Mycoplasma contamination	The cell lines were not tested for mycoplasma contamination.
Commonly misidentified lines (See ICLAC register)	No cell lines used in this study were commonly misidentified lines.
